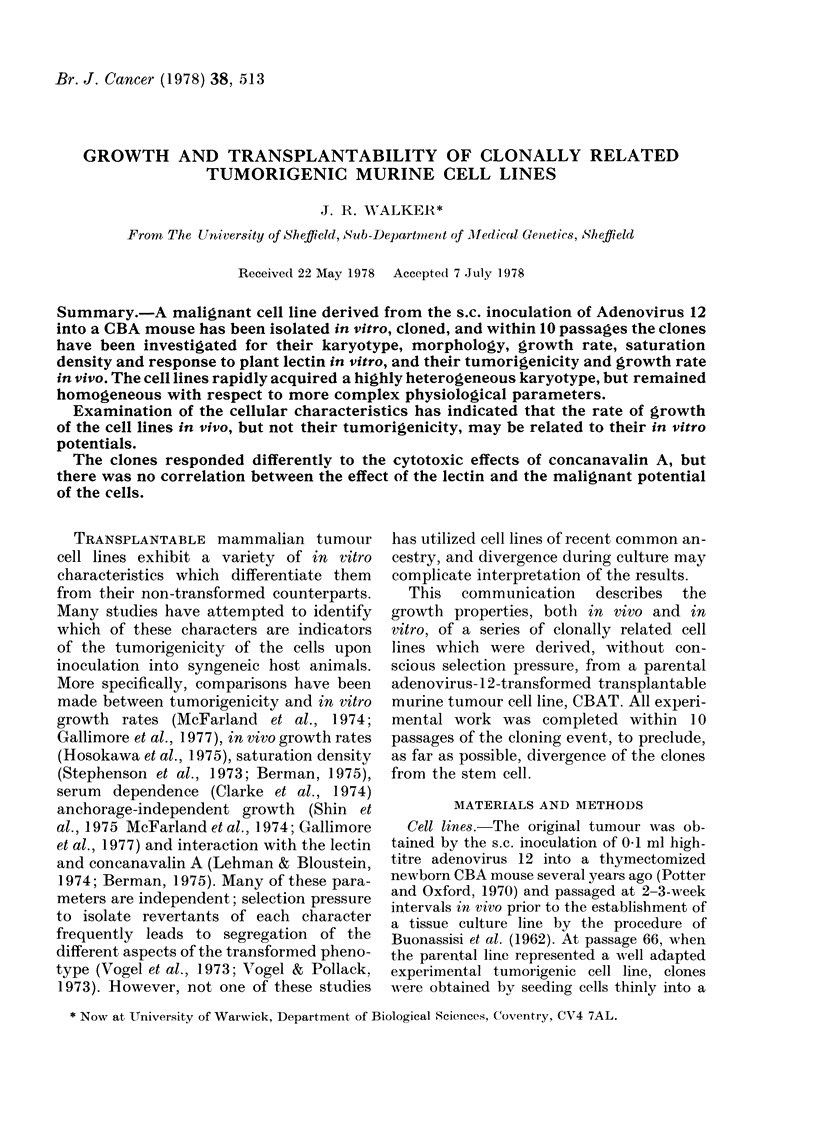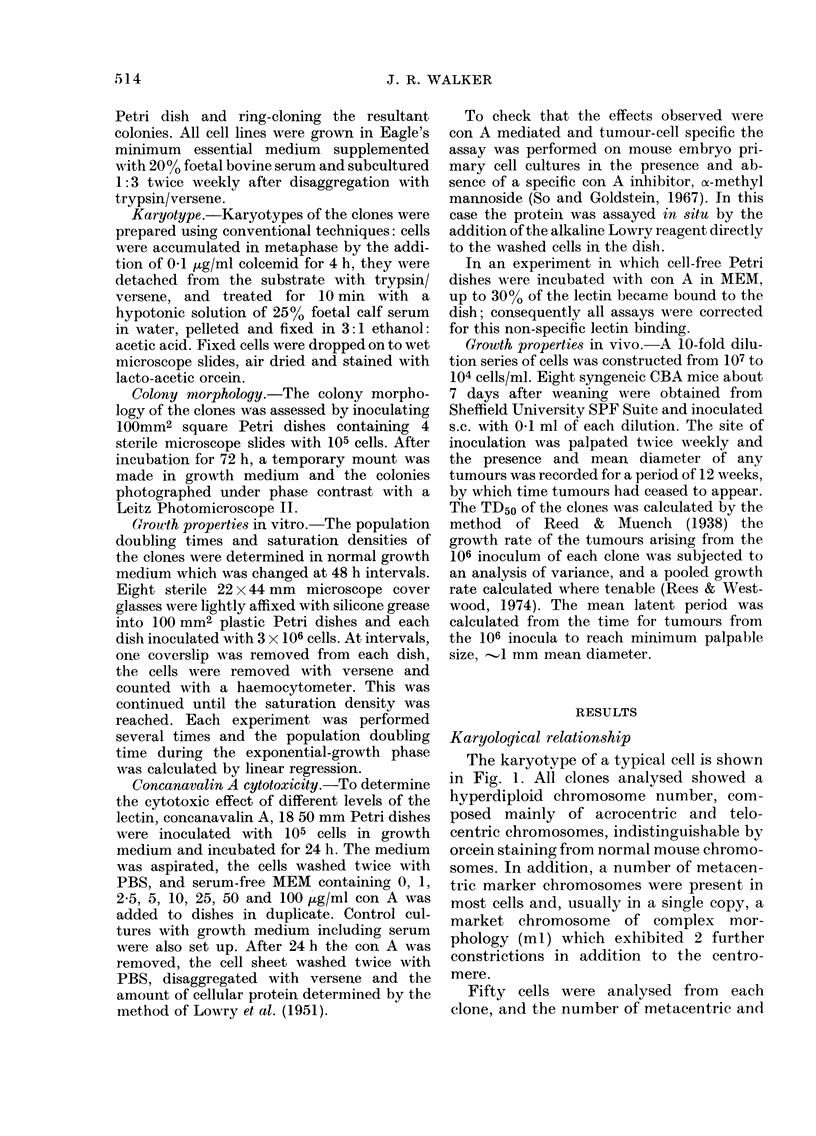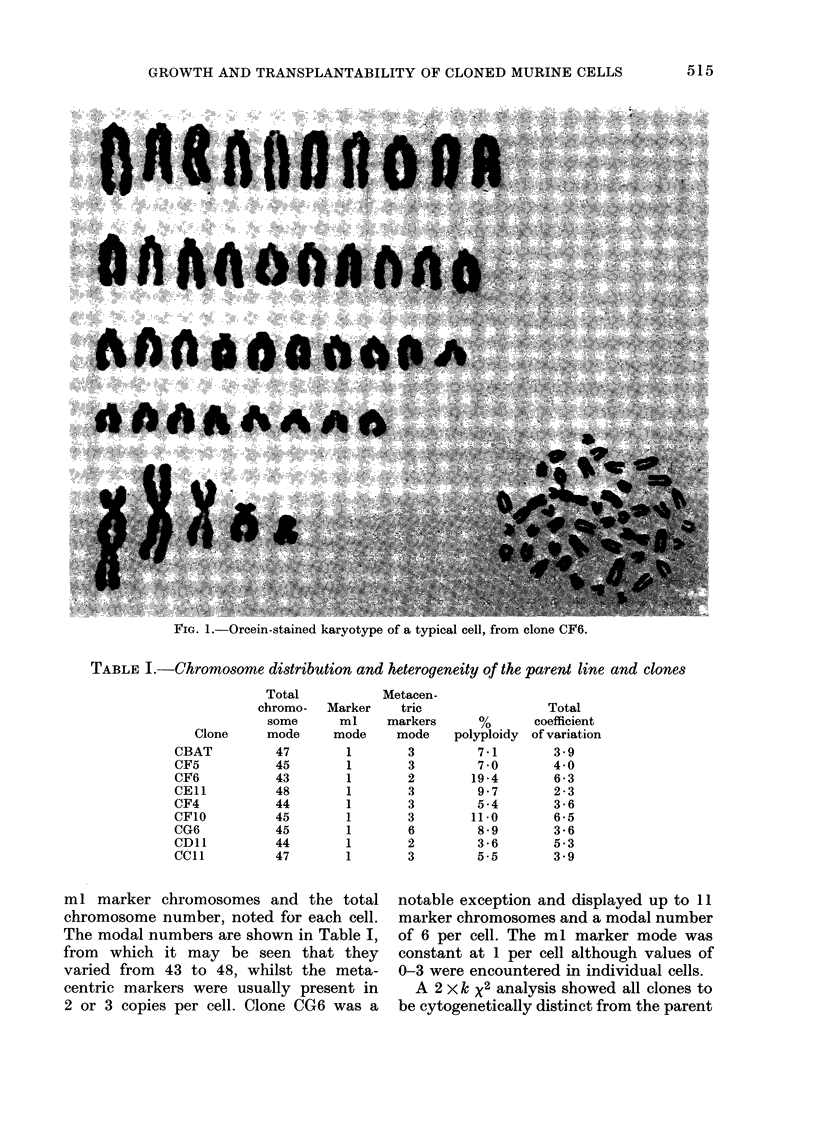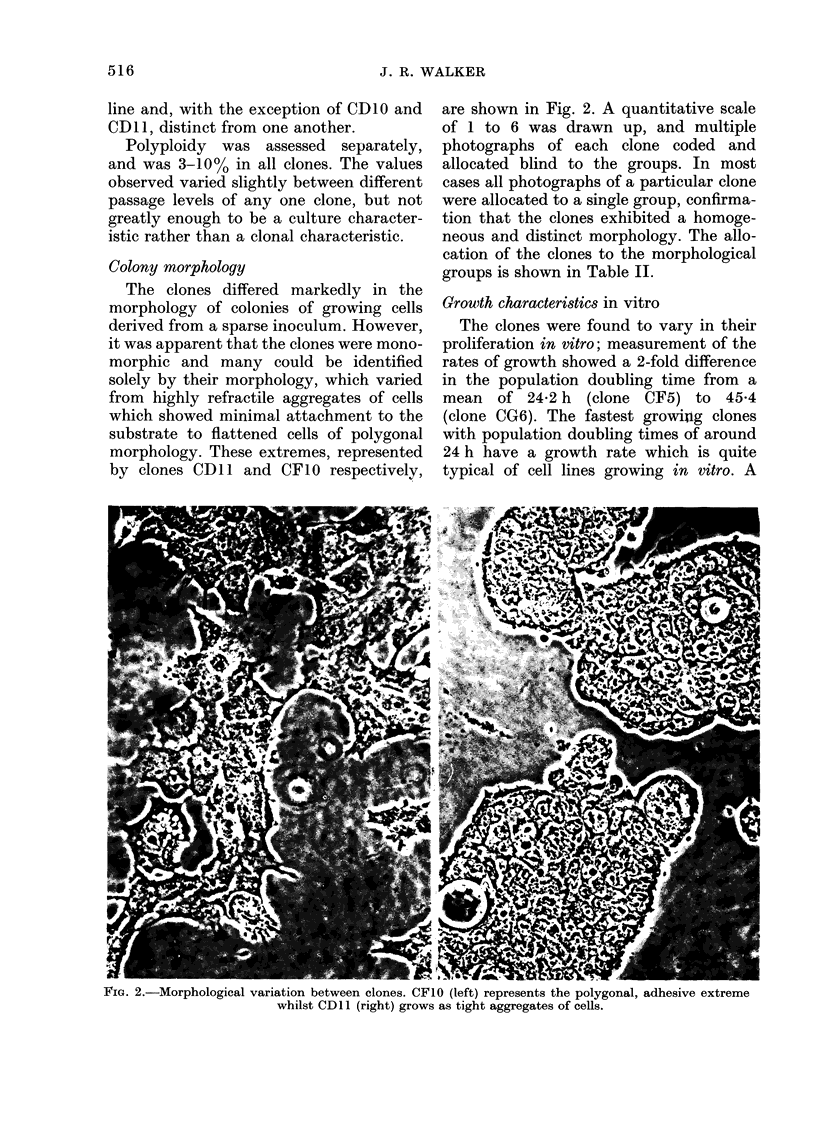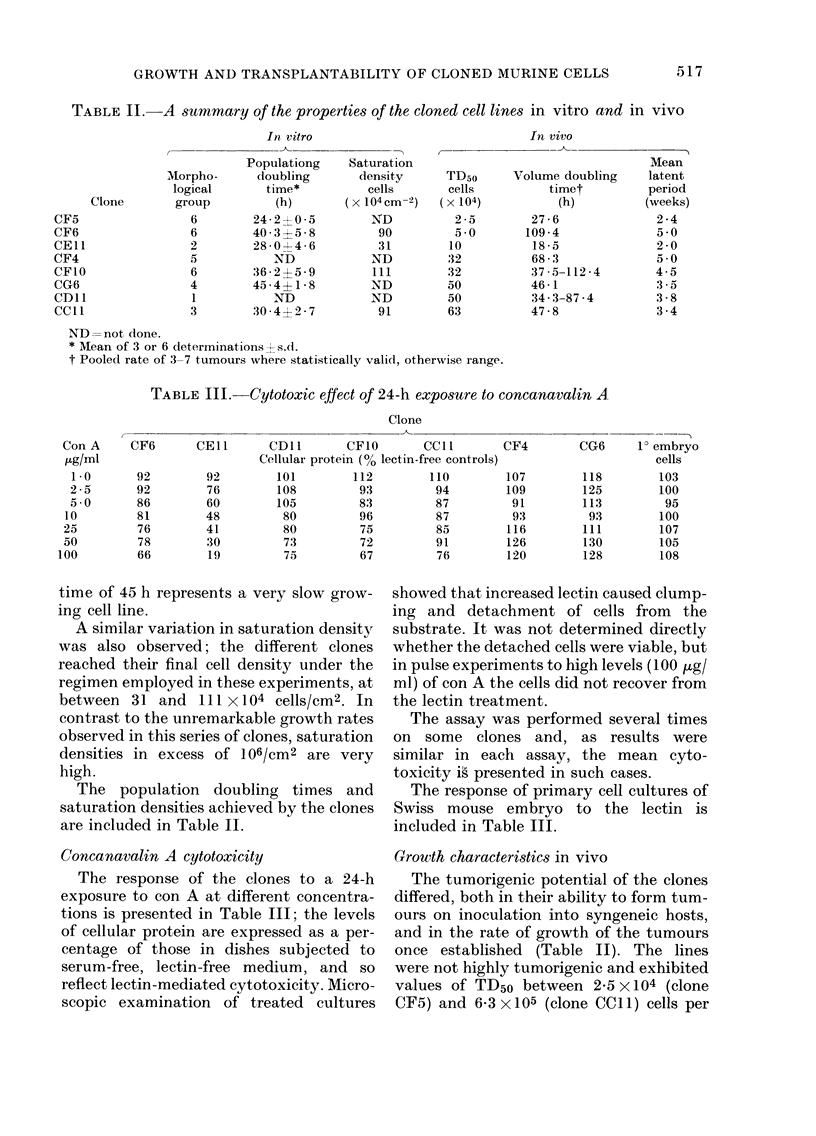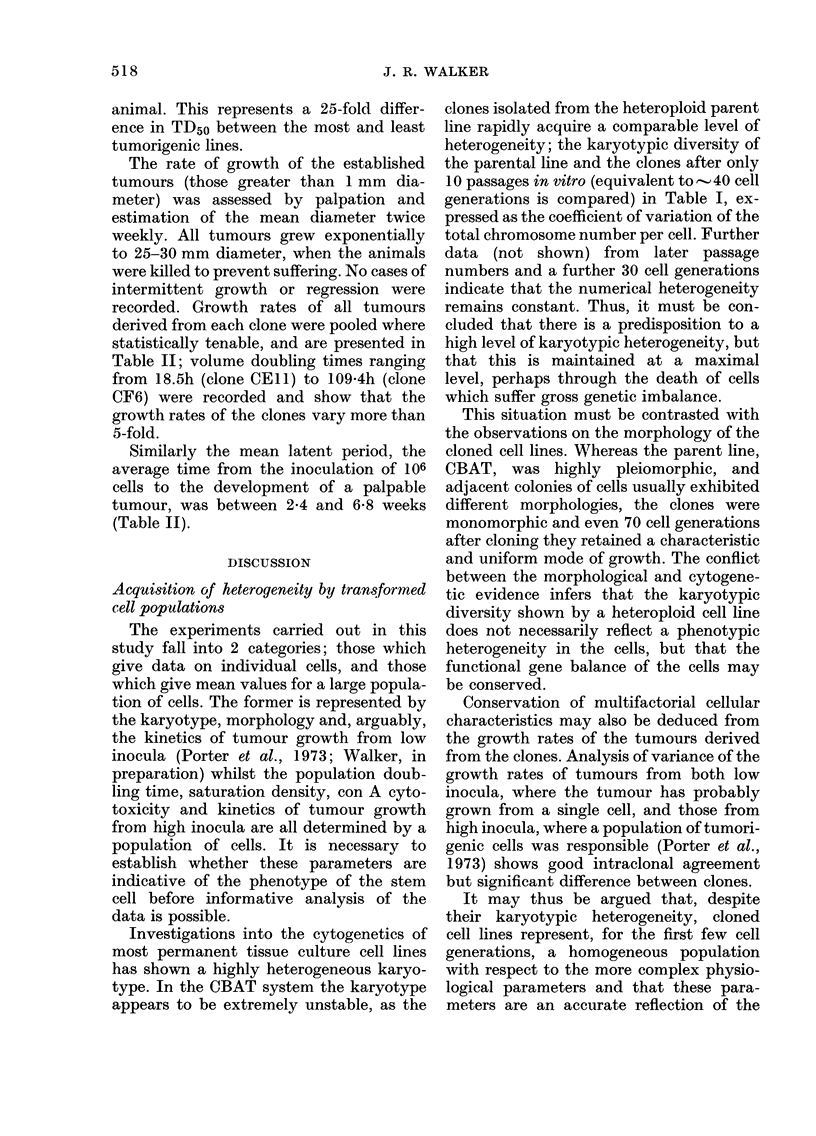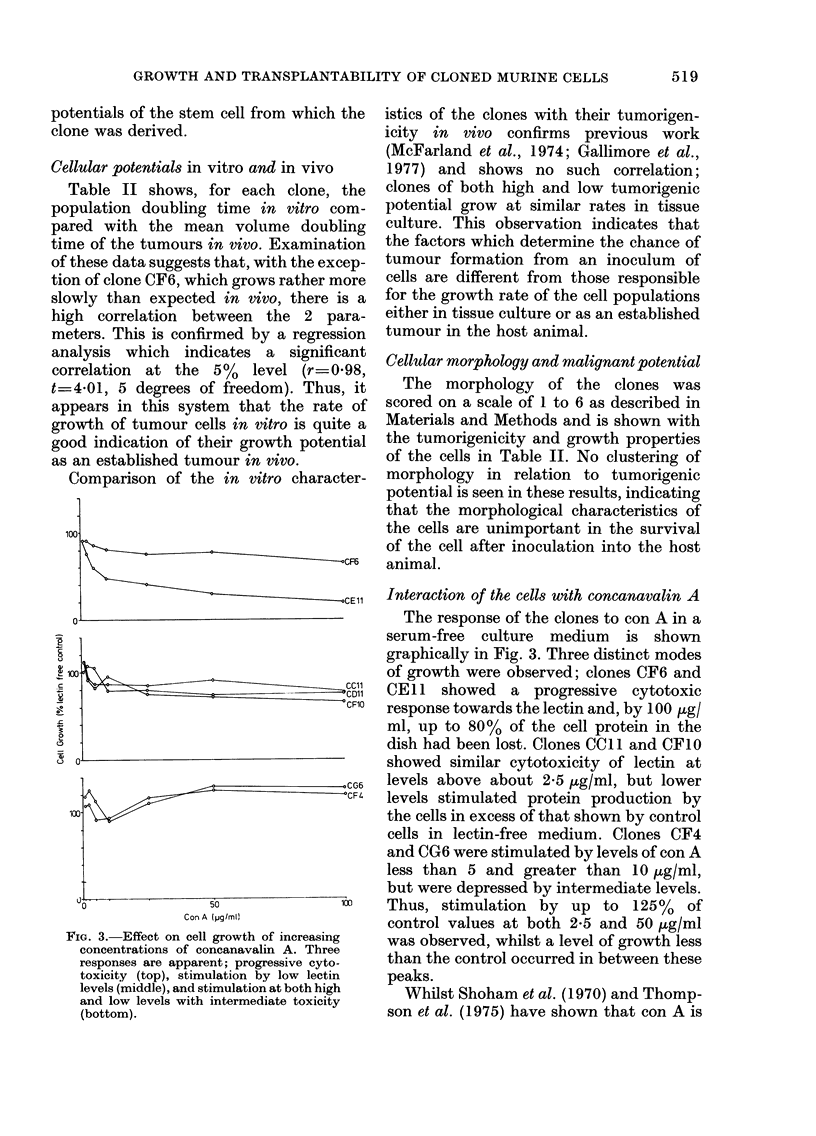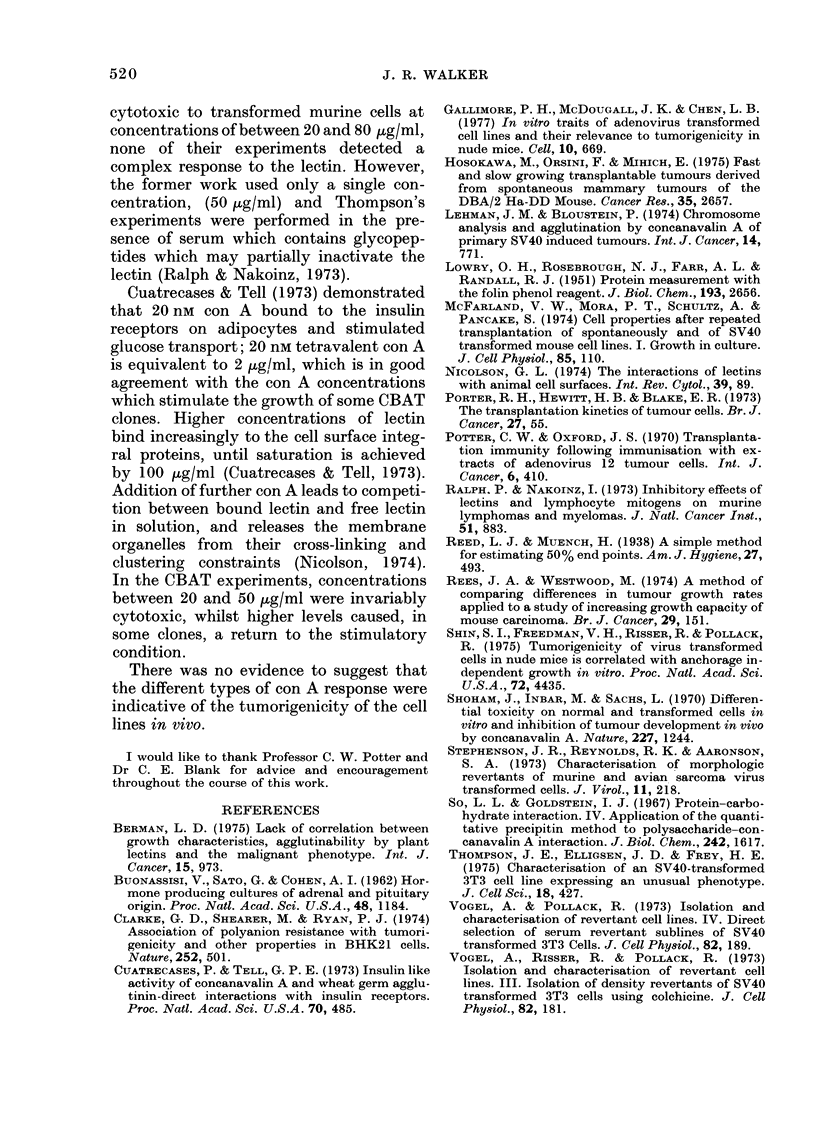# Growth and transplantability of clonally related tumorigenic murine cell lines.

**DOI:** 10.1038/bjc.1978.238

**Published:** 1978-10

**Authors:** J. R. Walker

## Abstract

**Images:**


*Br. J. Cancer* (1978) **38**, 513

### GROWTH AND TRANSPLANTABILITY OF CLONALLY RELATED TUMORIGENIC MURINE CELL LINES

J. R. WALKER*

*From The University of Sheffield, Sub-Department of Medical Genetics, Sheffield*

Received 22 May 1978 Accepted 7 July 1978

**Summary.—A malignant cell line derived from the s.c. inoculation of Adenovirus 12 into a CBA mouse has been isolated *in vitro*, cloned, and within 10 passages the clones have been investigated for their karyotype, morphology, growth rate, saturation density and response to plant lectin *in vitro*, and their tumorigenicity and growth rate *in vivo*. The cell lines rapidly acquired a highly heterogeneous karyotype, but remained homogeneous with respect to more complex physiological parameters.**

**Examination of the cellular characteristics has indicated that the rate of growth of the cell lines *in vivo*, but not their tumorigenicity, may be related to their *in vitro* potentials.**

**The clones responded differently to the cytotoxic effects of concanavalin A, but there was no correlation between the effect of the lectin and the malignant potential of the cells.**

TRANSPLANTABLE mammalian tumour cell lines exhibit a variety of *in vitro* characteristics which differentiate them from their non-transformed counterparts. Many studies have attempted to identify which of these characters are indicators of the tumorigenicity of the cells upon inoculation into syngeneic host animals. More specifically, comparisons have been made between tumorigenicity and *in vitro* growth rates (McFarland *et al.*, 1974; Gallimore *et al.*, 1977), *in vivo* growth rates (Hosokawa *et al.*, 1975), saturation density (Stephenson *et al.*, 1973; Berman, 1975), serum dependence (Clarke *et al.*, 1974) anchorage-independent growth (Shin *et al.*, 1975 McFarland *et al.*, 1974; Gallimore *et al.*, 1977) and interaction with the lectin and concanavalin A (Lehman & Bloustein, 1974; Berman, 1975). Many of these parameters are independent; selection pressure to isolate revertants of each character frequently leads to segregation of the different aspects of the transformed phenotype (Vogel *et al.*, 1973; Vogel & Pollack, 1973). However, not one of these studies has utilized cell lines of recent common ancestry, and divergence during culture may complicate interpretation of the results.

This communication describes the growth properties, both *in vivo* and *in vitro*, of a series of clonally related cell lines which were derived, without conscious selection pressure, from a parental adenovirus-12-transformed transplantable murine tumour cell line, CBAT. All experimental work was completed within 10 passages of the cloning event, to preclude, as far as possible, divergence of the clones from the stem cell.

### MATERIALS AND METHODS

*Cell lines.*—The original tumour was obtained by the s.c. inoculation of 0·1 ml high-titre adenovirus 12 into a thymectomized newborn CBA mouse several years ago (Potter and Oxford, 1970) and passaged at 2–3-week intervals *in vivo* prior to the establishment of a tissue culture line by the procedure of Buonassisi *et al.* (1962). At passage 66, when the parental line represented a well adapted experimental tumorigenic cell line, clones were obtained by seeding cells thinly into a

* Now at University of Warwick, Department of Biological Sciences, Coventry, CV4 7AL.

Petri dish and ring-cloning the resultant colonies. All cell lines were grown in Eagle's minimum essential medium supplemented with 20% foetal bovine serum and subcultured 1:3 twice weekly after disaggregation with trypsin/versene.

*Karyotype.*—Karyotypes of the clones were prepared using conventional techniques: cells were accumulated in metaphase by the addition of 0·1 μg/ml colcemid for 4 h, they were detached from the substrate with trypsin/versene, and treated for 10 min with a hypotonic solution of 25% foetal calf serum in water, pelleted and fixed in 3:1 ethanol: acetic acid. Fixed cells were dropped on to wet microscope slides, air dried and stained with lacto-acetic orcein.

*Colony morphology.*—The colony morphology of the clones was assessed by inoculating $100 mm^2$ square Petri dishes containing 4 sterile microscope slides with $10^5$ cells. After incubation for 72 h, a temporary mount was made in growth medium and the colonies photographed under phase contrast with a Leitz Photomicroscope II.

*Growth properties* in vitro.—The population doubling times and saturation densities of the clones were determined in normal growth medium which was changed at 48 h intervals. Eight sterile 22 × 44 mm microscope cover glasses were lightly affixed with silicone grease into $100 mm^2$ plastic Petri dishes and each dish inoculated with $3 \times 10^6$ cells. At intervals, one coverslip was removed from each dish, the cells were removed with versene and counted with a haemocytometer. This was continued until the saturation density was reached. Each experiment was performed several times and the population doubling time during the exponential-growth phase was calculated by linear regression.

*Concanavalin A cytotoxicity.*—To determine the cytotoxic effect of different levels of the lectin, concanavalin A, 18 50 mm Petri dishes were inoculated with $10^5$ cells in growth medium and incubated for 24 h. The medium was aspirated, the cells washed twice with PBS, and serum-free MEM containing 0, 1, 2·5, 5, 10, 25, 50 and 100 μg/ml con A was added to dishes in duplicate. Control cultures with growth medium including serum were also set up. After 24 h the con A was removed, the cell sheet washed twice with PBS, disaggregated with versene and the amount of cellular protein determined by the method of Lowry *et al.* (1951).

To check that the effects observed were con A mediated and tumour-cell specific the assay was performed on mouse embryo primary cell cultures in the presence and absence of a specific con A inhibitor, α-methyl mannoside (So and Goldstein, 1967). In this case the protein was assayed *in situ* by the addition of the alkaline Lowry reagent directly to the washed cells in the dish.

In an experiment in which cell-free Petri dishes were incubated with con A in MEM, up to 30% of the lectin became bound to the dish; consequently all assays were corrected for this non-specific lectin binding.

*Growth properties* in vivo.—A 10-fold dilution series of cells was constructed from $10^7$ to $10^4$ cells/ml. Eight syngeneic CBA mice about 7 days after weaning were obtained from Sheffield University SPF Suite and inoculated s.c. with 0·1 ml of each dilution. The site of inoculation was palpated twice weekly and the presence and mean diameter of any tumours was recorded for a period of 12 weeks, by which time tumours had ceased to appear. The $TD_{50}$ of the clones was calculated by the method of Reed & Muench (1938) the growth rate of the tumours arising from the $10^6$ inoculum of each clone was subjected to an analysis of variance, and a pooled growth rate calculated where tenable (Rees & Westwood, 1974). The mean latent period was calculated from the time for tumours from the $10^6$ inocula to reach minimum palpable size, ~1 mm mean diameter.

### RESULTS

### *Karyological relationship*

The karyotype of a typical cell is shown in Fig. 1. All clones analysed showed a hyperdiploid chromosome number, composed mainly of acrocentric and telocentric chromosomes, indistinguishable by orcein staining from normal mouse chromosomes. In addition, a number of metacentric marker chromosomes were present in most cells and, usually in a single copy, a market chromosome of complex morphology (m1) which exhibited 2 further constrictions in addition to the centromere.

Fifty cells were analysed from each clone, and the number of metacentric and

FIG. 1.—Orcein-stained karyotype of a typical cell, from clone CF6.

TABLE I.—*Chromosome distribution and heterogeneity of the parent line and clones*

| Clone | Total chromosome mode | Marker ml mode | Metacentric markers mode | % polyploidy | Total coefficient of variation |
|---|---|---|---|---|---|
| CBAT | 47 | 1 | 3 | 7·1 | 3·9 |
| CF5 | 45 | 1 | 3 | 7·0 | 4·0 |
| CF6 | 43 | 1 | 2 | 19·4 | 6·3 |
| CE11 | 48 | 1 | 3 | 9·7 | 2·3 |
| CF4 | 44 | 1 | 3 | 5·4 | 3·6 |
| CF10 | 45 | 1 | 3 | 11·0 | 6·5 |
| CG6 | 45 | 1 | 6 | 8·9 | 3·6 |
| CD11 | 44 | 1 | 2 | 3·6 | 5·3 |
| CC11 | 47 | 1 | 3 | 5·5 | 3·9 |

ml marker chromosomes and the total chromosome number, noted for each cell. The modal numbers are shown in Table I, from which it may be seen that they varied from 43 to 48, whilst the metacentric markers were usually present in 2 or 3 copies per cell. Clone CG6 was a notable exception and displayed up to 11 marker chromosomes and a modal number of 6 per cell. The ml marker mode was constant at 1 per cell although values of 0–3 were encountered in individual cells.

A $2 \times k$ $\chi^2$ analysis showed all clones to be cytogenetically distinct from the parent line and, with the exception of CD10 and CD11, distinct from one another.

Polyploidy was assessed separately, and was 3–10% in all clones. The values observed varied slightly between different passage levels of any one clone, but not greatly enough to be a culture characteristic rather than a clonal characteristic.

*Colony morphology*

The clones differed markedly in the morphology of colonies of growing cells derived from a sparse inoculum. However, it was apparent that the clones were monomorphic and many could be identified solely by their morphology, which varied from highly refractile aggregates of cells which showed minimal attachment to the substrate to flattened cells of polygonal morphology. These extremes, represented by clones CD11 and CF10 respectively, are shown in Fig. 2. A quantitative scale of 1 to 6 was drawn up, and multiple photographs of each clone coded and allocated blind to the groups. In most cases all photographs of a particular clone were allocated to a single group, confirmation that the clones exhibited a homogeneous and distinct morphology. The allocation of the clones to the morphological groups is shown in Table II.

*Growth characteristics* in vitro

The clones were found to vary in their proliferation *in vitro*; measurement of the rates of growth showed a 2-fold difference in the population doubling time from a mean of 24·2 h (clone CF5) to 45·4 (clone CG6). The fastest growing clones with population doubling times of around 24 h have a growth rate which is quite typical of cell lines growing *in vitro*. A

FIG. 2.—Morphological variation between clones. CF10 (left) represents the polygonal, adhesive extreme whilst CD11 (right) grows as tight aggregates of cells.

TABLE II.—*A summary of the properties of the cloned cell lines* in vitro *and* in vivo

| | *In vitro* | | | *In vivo* | | |
|---|---|---|---|---|---|---|
| Clone | Morphological group | Populationg doubling time* (h) | Saturation density cells (× $10^4$ cm$^{-2}$) | $TD_{50}$ cells (× $10^4$) | Volume doubling time† (h) | Mean latent period (weeks) |
| CF5 | 6 | 24·2±0·5 | ND | 2·5 | 27·6 | 2·4 |
| CF6 | 6 | 40·3±5·8 | 90 | 5·0 | 109·4 | 5·0 |
| CE11 | 2 | 28·0±4·6 | 31 | 10 | 18·5 | 2·0 |
| CF4 | 5 | ND | ND | 32 | 68·3 | 5·0 |
| CF10 | 6 | 36·2±5·9 | 111 | 32 | 37·5–112·4 | 4·5 |
| CG6 | 4 | 45·4±1·8 | ND | 50 | 46·1 | 3·5 |
| CD11 | 1 | ND | ND | 50 | 34·3–87·4 | 3·8 |
| CC11 | 3 | 30·4±2·7 | 91 | 63 | 47·8 | 3·4 |

ND = not done.
* Mean of 3 or 6 determinations ± s.d.
† Pooled rate of 3–7 tumours where statistically valid, otherwise range.

TABLE III.—*Cytotoxic effect of* 24-h *exposure to concanavalin A*

| | Clone | | | | | | | |
|---|---|---|---|---|---|---|---|---|
| Con A μg/ml | CF6 | CE11 | CD11 | CF10 | CC11 | CF4 | CG6 | 1° embryo cells |
| | Cellular protein (% lectin-free controls) | | | | | | | |
| 1·0 | 92 | 92 | 101 | 112 | 110 | 107 | 118 | 103 |
| 2·5 | 92 | 76 | 108 | 93 | 94 | 109 | 125 | 100 |
| 5·0 | 86 | 60 | 105 | 83 | 87 | 91 | 113 | 95 |
| 10 | 81 | 48 | 80 | 96 | 87 | 93 | 93 | 100 |
| 25 | 76 | 41 | 80 | 75 | 85 | 116 | 111 | 107 |
| 50 | 78 | 30 | 73 | 72 | 91 | 126 | 130 | 105 |
| 100 | 66 | 19 | 75 | 67 | 76 | 120 | 128 | 108 |

time of 45 h represents a very slow growing cell line.

A similar variation in saturation density was also observed; the different clones reached their final cell density under the regimen employed in these experiments, at between 31 and $111 \times 10^4$ cells/cm$^2$. In contrast to the unremarkable growth rates observed in this series of clones, saturation densities in excess of $10^6$/cm$^2$ are very high.

The population doubling times and saturation densities achieved by the clones are included in Table II.

*Concanavalin A cytotoxicity*

The response of the clones to a 24-h exposure to con A at different concentrations is presented in Table III; the levels of cellular protein are expressed as a percentage of those in dishes subjected to serum-free, lectin-free medium, and so reflect lectin-mediated cytotoxicity. Microscopic examination of treated cultures showed that increased lectin caused clumping and detachment of cells from the substrate. It was not determined directly whether the detached cells were viable, but in pulse experiments to high levels (100 μg/ml) of con A the cells did not recover from the lectin treatment.

The assay was performed several times on some clones and, as results were similar in each assay, the mean cytotoxicity is presented in such cases.

The response of primary cell cultures of Swiss mouse embryo to the lectin is included in Table III.

*Growth characteristics* in vivo

The tumorigenic potential of the clones differed, both in their ability to form tumours on inoculation into syngeneic hosts, and in the rate of growth of the tumours once established (Table II). The lines were not highly tumorigenic and exhibited values of $TD_{50}$ between $2{\cdot}5 \times 10^4$ (clone CF5) and $6{\cdot}3 \times 10^5$ (clone CC11) cells per animal. This represents a 25-fold difference in $TD_{50}$ between the most and least tumorigenic lines.

The rate of growth of the established tumours (those greater than 1 mm diameter) was assessed by palpation and estimation of the mean diameter twice weekly. All tumours grew exponentially to 25–30 mm diameter, when the animals were killed to prevent suffering. No cases of intermittent growth or regression were recorded. Growth rates of all tumours derived from each clone were pooled where statistically tenable, and are presented in Table II; volume doubling times ranging from 18.5h (clone CE11) to 109·4h (clone CF6) were recorded and show that the growth rates of the clones vary more than 5-fold.

Similarly the mean latent period, the average time from the inoculation of $10^6$ cells to the development of a palpable tumour, was between 2·4 and 6·8 weeks (Table II).

### DISCUSSION

### *Acquisition of heterogeneity by transformed cell populations*

The experiments carried out in this study fall into 2 categories; those which give data on individual cells, and those which give mean values for a large population of cells. The former is represented by the karyotype, morphology and, arguably, the kinetics of tumour growth from low inocula (Porter *et al.*, 1973; Walker, in preparation) whilst the population doubling time, saturation density, con A cytotoxicity and kinetics of tumour growth from high inocula are all determined by a population of cells. It is necessary to establish whether these parameters are indicative of the phenotype of the stem cell before informative analysis of the data is possible.

Investigations into the cytogenetics of most permanent tissue culture cell lines has shown a highly heterogeneous karyotype. In the CBAT system the karyotype appears to be extremely unstable, as the clones isolated from the heteroploid parent line rapidly acquire a comparable level of heterogeneity; the karyotypic diversity of the parental line and the clones after only 10 passages *in vitro* (equivalent to $\sim$40 cell generations is compared) in Table I, expressed as the coefficient of variation of the total chromosome number per cell. Further data (not shown) from later passage numbers and a further 30 cell generations indicate that the numerical heterogeneity remains constant. Thus, it must be concluded that there is a predisposition to a high level of karyotypic heterogeneity, but that this is maintained at a maximal level, perhaps through the death of cells which suffer gross genetic imbalance.

This situation must be contrasted with the observations on the morphology of the cloned cell lines. Whereas the parent line, CBAT, was highly pleiomorphic, and adjacent colonies of cells usually exhibited different morphologies, the clones were monomorphic and even 70 cell generations after cloning they retained a characteristic and uniform mode of growth. The conflict between the morphological and cytogenetic evidence infers that the karyotypic diversity shown by a heteroploid cell line does not necessarily reflect a phenotypic heterogeneity in the cells, but that the functional gene balance of the cells may be conserved.

Conservation of multifactorial cellular characteristics may also be deduced from the growth rates of the tumours derived from the clones. Analysis of variance of the growth rates of tumours from both low inocula, where the tumour has probably grown from a single cell, and those from high inocula, where a population of tumorigenic cells was responsible (Porter *et al.*, 1973) shows good intraclonal agreement but significant difference between clones.

It may thus be argued that, despite their karyotypic heterogeneity, cloned cell lines represent, for the first few cell generations, a homogeneous population with respect to the more complex physiological parameters and that these parameters are an accurate reflection of the potentials of the stem cell from which the clone was derived.

*Cellular potentials* in vitro *and* in vivo

Table II shows, for each clone, the population doubling time *in vitro* compared with the mean volume doubling time of the tumours *in vivo*. Examination of these data suggests that, with the exception of clone CF6, which grows rather more slowly than expected *in vivo*, there is a high correlation between the 2 parameters. This is confirmed by a regression analysis which indicates a significant correlation at the 5% level ($r=0{\cdot}98$, $t=4{\cdot}01$, 5 degrees of freedom). Thus, it appears in this system that the rate of growth of tumour cells *in vitro* is quite a good indication of their growth potential as an established tumour *in vivo*.

Comparison of the *in vitro* characteristics of the clones with their tumorigenicity *in vivo* confirms previous work (McFarland *et al.*, 1974; Gallimore *et al.*, 1977) and shows no such correlation; clones of both high and low tumorigenic potential grow at similar rates in tissue culture. This observation indicates that the factors which determine the chance of tumour formation from an inoculum of cells are different from those responsible for the growth rate of the cell populations either in tissue culture or as an established tumour in the host animal.

FIG. 3.—Effect on cell growth of increasing concentrations of concanavalin A. Three responses are apparent; progressive cytotoxicity (top), stimulation by low lectin levels (middle), and stimulation at both high and low levels with intermediate toxicity (bottom).

*Cellular morphology and malignant potential*

The morphology of the clones was scored on a scale of 1 to 6 as described in Materials and Methods and is shown with the tumorigenicity and growth properties of the cells in Table II. No clustering of morphology in relation to tumorigenic potential is seen in these results, indicating that the morphological characteristics of the cells are unimportant in the survival of the cell after inoculation into the host animal.

*Interaction of the cells with concanavalin A*

The response of the clones to con A in a serum-free culture medium is shown graphically in Fig. 3. Three distinct modes of growth were observed; clones CF6 and CE11 showed a progressive cytotoxic response towards the lectin and, by 100 μg/ml, up to 80% of the cell protein in the dish had been lost. Clones CC11 and CF10 showed similar cytotoxicity of lectin at levels above about 2·5 μg/ml, but lower levels stimulated protein production by the cells in excess of that shown by control cells in lectin-free medium. Clones CF4 and CG6 were stimulated by levels of con A less than 5 and greater than 10 μg/ml, but were depressed by intermediate levels. Thus, stimulation by up to 125% of control values at both 2·5 and 50 μg/ml was observed, whilst a level of growth less than the control occurred in between these peaks.

Whilst Shoham *et al.* (1970) and Thompson *et al.* (1975) have shown that con A is cytotoxic to transformed murine cells at concentrations of between 20 and 80 $\mu$g/ml, none of their experiments detected a complex response to the lectin. However, the former work used only a single concentration, (50 $\mu$g/ml) and Thompson's experiments were performed in the presence of serum which contains glycopeptides which may partially inactivate the lectin (Ralph & Nakoinz, 1973).

Cuatrecases & Tell (1973) demonstrated that 20 nM con A bound to the insulin receptors on adipocytes and stimulated glucose transport; 20 nM tetravalent con A is equivalent to 2 $\mu$g/ml, which is in good agreement with the con A concentrations which stimulate the growth of some CBAT clones. Higher concentrations of lectin bind increasingly to the cell surface integral proteins, until saturation is achieved by 100 $\mu$g/ml (Cuatrecases & Tell, 1973). Addition of further con A leads to competition between bound lectin and free lectin in solution, and releases the membrane organelles from their cross-linking and clustering constraints (Nicolson, 1974). In the CBAT experiments, concentrations between 20 and 50 $\mu$g/ml were invariably cytotoxic, whilst higher levels caused, in some clones, a return to the stimulatory condition.

There was no evidence to suggest that the different types of con A response were indicative of the tumorigenicity of the cell lines *in vivo*.

I would like to thank Professor C. W. Potter and Dr C. E. Blank for advice and encouragement throughout the course of this work.

### REFERENCES

BERMAN, L. D. (1975) Lack of correlation between growth characteristics, agglutinability by plant lectins and the malignant phenotype. *Int. J. Cancer*, **15**, 973.

BUONASSISI, V., SATO, G. & COHEN, A. I. (1962) Hormone producing cultures of adrenal and pituitary origin. *Proc. Natl. Acad. Sci. U.S.A.*, **48**, 1184.

CLARKE, G. D., SHEARER, M. & RYAN, P. J. (1974) Association of polyanion resistance with tumorigenicity and other properties in BHK21 cells. *Nature*, **252**, 501.

CUATRECASES, P. & TELL, G. P. E. (1973) Insulin like activity of concanavalin A and wheat germ agglutinin-direct interactions with insulin receptors. *Proc. Natl. Acad. Sci. U.S.A.* **70**, 485.

GALLIMORE, P. H., McDOUGALL, J. K. & CHEN, L. B. (1977) *In vitro* traits of adenovirus transformed cell lines and their relevance to tumorigenicity in nude mice. *Cell*, **10**, 669.

HOSOKAWA, M., ORSINI, F. & MIHICH, E. (1975) Fast and slow growing transplantable tumours derived from spontaneous mammary tumours of the DBA/2 Ha-DD Mouse. *Cancer Res.*, **35**, 2657.

LEHMAN, J. M. & BLOUSTEIN, P. (1974) Chromosome analysis and agglutination by concanavalin A of primary SV40 induced tumours. *Int. J. Cancer*, **14**, 771.

LOWRY, O. H., ROSEBROUGH, N. J., FARR, A. L. & RANDALL, R. J. (1951) Protein measurement with the folin phenol reagent. *J. Biol. Chem.*, **193**, 2656.

McFARLAND, V. W., MORA, P. T., SCHULTZ, A. & PANCAKE, S. (1974) Cell properties after repeated transplantation of spontaneously and of SV40 transformed mouse cell lines. I. Growth in culture. *J. Cell Physiol.*, **85**, 110.

NICOLSON, G. L. (1974) The interactions of lectins with animal cell surfaces. *Int. Rev. Cytol.*, **39**, 89.

PORTER, R. H., HEWITT, H. B. & BLAKE, E. R. (1973) The transplantation kinetics of tumour cells. *Br. J. Cancer*, **27**, 55.

POTTER, C. W. & OXFORD, J. S. (1970) Transplantation immunity following immunisation with extracts of adenovirus 12 tumour cells. *Int. J. Cancer*, **6**, 410.

RALPH, P. & NAKOINZ, I. (1973) Inhibitory effects of lectins and lymphocyte mitogens on murine lymphomas and myelomas. *J. Natl. Cancer Inst.*, **51**, 883.

REED, L. J. & MUENCH, H. (1938) A simple method for estimating 50% end points. *Am. J. Hygiene*, **27**, 493.

REES, J. A. & WESTWOOD, M. (1974) A method of comparing differences in tumour growth rates applied to a study of increasing growth capacity of mouse carcinoma. *Br. J. Cancer*, **29**, 151.

SHIN, S. I., FREEDMAN, V. H., RISSER, R. & POLLACK, R. (1975) Tumorigenicity of virus transformed cells in nude mice is correlated with anchorage independent growth *in vitro*. *Proc. Natl. Acad. Sci. U.S.A.*, **72**, 4435.

SHOHAM, J., INBAR, M. & SACHS, L. (1970) Differential toxicity on normal and transformed cells *in vitro* and inhibition of tumour development *in vivo* by concanavalin A. *Nature*, **227**, 1244.

STEPHENSON, J. R., REYNOLDS, R. K. & AARONSON, S. A. (1973) Characterisation of morphologic revertants of murine and avian sarcoma virus transformed cells. *J. Virol.*, **11**, 218.

SO, L. L. & GOLDSTEIN, I. J. (1967) Protein–carbohydrate interaction. IV. Application of the quantitative precipitin method to polysaccharide–concanavalin A interaction. *J. Biol. Chem.*, **242**, 1617.

THOMPSON, J. E., ELLIGSEN, J. D. & FREY, H. E. (1975) Characterisation of an SV40-transformed 3T3 cell line expressing an unusual phenotype. *J. Cell Sci.*, **18**, 427.

VOGEL, A. & POLLACK, R. (1973) Isolation and characterisation of revertant cell lines. IV. Direct selection of serum revertant sublines of SV40 transformed 3T3 Cells. *J. Cell Physiol.*, **82**, 189.

VOGEL, A., RISSER, R. & POLLACK, R. (1973) Isolation and characterisation of revertant cell lines. III. Isolation of density revertants of SV40 transformed 3T3 cells using colchicine. *J. Cell Physiol.*, **82**, 181.